# Mutation Analysis of Colorectal and Gastric Carcinomas Originating from Adenomas: Insights into Genomic Evolution Associated with Malignant Progression

**DOI:** 10.3390/cancers12020325

**Published:** 2020-01-31

**Authors:** Sung Hak Lee, Jinseon Yoo, Young Soo Song, Chul-Hyun Lim, Tae-Min Kim

**Affiliations:** 1 Department of Hospital Pathology, Seoul St. Mary’s Hospital, College of Medicine, The Catholic University of Korea, 222 Banpodae-ro, Seocho-gu, Seoul 06591, Korea; hakjjang@catholic.ac.kr; 2 Department of Medical Informatics, The Catholic University of Korea, 222 Banpodae-ro, Seocho-gu, Seoul 06591, Korea; dbwlstjs0@gmail.com; 3 Cancer Research Institute, College of Medicine, The Catholic University of Korea, 222 Banpodae-ro, Seocho-gu, Seoul 06591, Korea; 4 Department of Pathology, College of Medicine, Konyang University, 158 Gwanjeodong-ro, Seo-gu, Daejeon 35365, Korea; lifen@hanyang.ac.kr; 5 Division of Gastroenterology, Department of Internal Medicine, Eunpyeong St. Mary’s Hospital, College of Medicine, The Catholic University of Korea, 1021, Tongil-ro, Eunpyeong-gu, Seoul 03312, Korea

**Keywords:** colorectal cancers, gastric cancers, somatic mutations, malignant progression, exome sequencing

## Abstract

Small malignant tumor foci arising from benign lesions are rare but offer a unique opportunity to investigate the genomic evolution that occurs during malignant transformation. In this study, we analyzed 11 colorectal and 10 gastric adenoma–carcinoma pairs, each of which represented malignant tumors (carcinomas) embedded in benign lesions (adenomas) found in the same patient. Whole-exome sequencing revealed that mutation abundance was variable across different cases, but comparable between adenoma–carcinoma pairs. When mutations were classified as adenoma-specific, carcinoma-specific, or common, adenoma-specific mutations were more enriched with subclonal mutations than were carcinoma-specific mutations, indicative of a perturbation in mutational subclonal architecture (such as selective sweep) during malignant transformation. Among the recurrent mutations in colorectal cancers, *APC* and *KRAS* mutations were common between adenomas and carcinomas, indicative of their early occurrence during genomic evolution. *TP53* mutations were often observed as adenoma-specific and therefore likely not associated with the emergence of malignant clones. Clonality-based enrichment analysis revealed that subclonal mutations of extracellular matrix genes in adenomas are more likely to be clonal in carcinomas, indicating potential roles for these genes in malignant transformation. Compared with colorectal cancers, gastric cancers showed more lesion-specific mutations than common mutations and higher levels of discordance in copy number profiles between matched adenomas and carcinomas, which may explain the elevated evolutionary dynamics and heterogeneity of gastric cancers compared to colorectal cancers. Taken together, this study demonstrates that co-existing benign and malignant lesions enable the evolution-based categorization of genomic alterations that may reveal clinically important biomarkers in colorectal and gastric cancers.

## 1. Introduction

Colorectal and gastric cancers are common gastrointestinal tumors worldwide representing major causes of cancer-related morbidity and mortality [1,2]. Endoscope-based screening has been useful in reducing the overall disease risk through detecting early stage diseases. However, at an advanced stage these cancers are largely incurable, with limited therapeutic options available. Large-scale cancer genome sequencing projects such as the Cancer Genome Atlas consortium have generated mutation profiles for thousands of colorectal and gastric cancers, leading to previously unrecognized driver mutations, perturbed regulatory programs, and distinct molecular taxa of the disease [3,4]. However, the investigation of genomic snapshots from fully transformed tumors across unrelated patients may be limited in gaining evolutionary insights. The acquisition of multiple biopsies for longitudinal cancer genome analysis is also largely limited for solid tumors.

During the multistep evolution of colorectal cancers, normal epithelial cells go through a series of well-defined histological stages, from dysplasia to adenoma and eventually through to carcinoma with metastases [5,6]. This stepwise evolution of cancer has been a widely accepted model for colorectal cancers and gastric cancers [7]. This model postulates that the growth of malignant tumors is followed by a selective sweep of the preceding lesions (such as adenomas) and thus, either benign adenomas or malignant carcinomas are observed at the time of biopsy or surgical resection. However, small malignant tumor foci are sometimes observed within residual adenomas [8]. Such histological representations—malignant tumor foci embedded in adenomatous lesions—represent two evolutionarily distinct states with a shared ancestry, offering an opportunity to investigate the genetic evolution underlying malignant transformation.

It is widely accepted that the histological progression of tumors occurs in parallel with the accumulation of genomic aberrations, some of which play roles in malignant progression. Recent cancer genome analyses have compared multiple specimens from a single individual to gain insights into the evolutionary history of cancer genomes [9]. For example, primary tumor genomes have been compared with matched, metastatic tumor genomes using genomic variants as evolutionary markers [10]. The acquisition of premalignant or benign tumor genomes along with their matched malignant tumor genomes is largely limited. The analysis of multiple, synchronous colorectal crypts has been proposed [11], but it is not clear whether the synchronous lesions, often physically separated in the organs, originate from the same ancestor or represent independent tumor foci.

In this study, we performed whole-exome sequencing of 11 colorectal and 10 gastric pairs of adenoma–carcinoma, all of which were obtained from histologically-confirmed invasive carcinomas arising from benign adenomatous lesions.

## 2. Results

### 2.1. Whole-Exome Sequencing and Somatic Variants

We performed whole-exome sequencing of paired benign and malignant lesions, consistent with carcinomas arising from adenomas, for 11 colorectal and 10 gastric cancers. The clinicopathological information of 21 patients is shown in Table S1.

**Figure 1:**
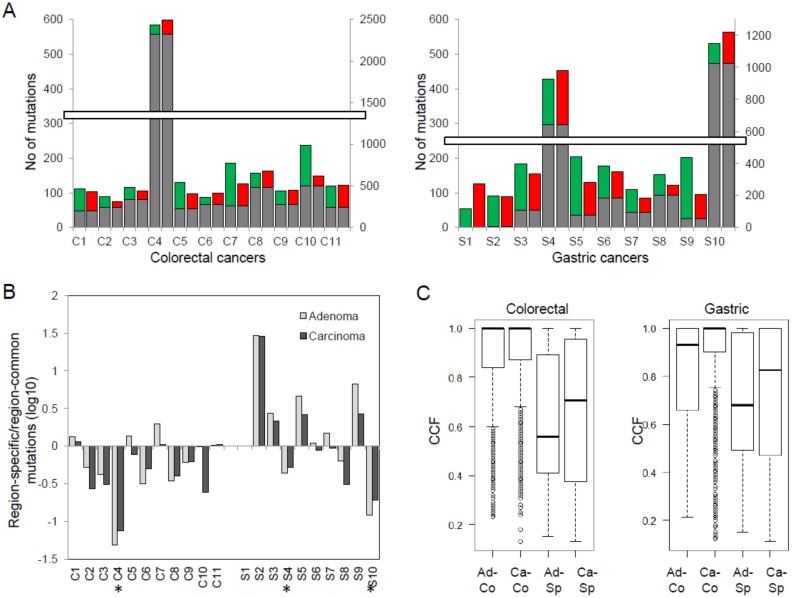
Exonic mutations of colorectal and gastric pairs of adenoma and carcinoma. (**A**) Mutational abundance (i.e., the number of exonic mutations) is shown for 21 pairs of adenoma and carcinoma (11 colorectal and 10 gastric; left and right, respectively). Lesion-common (gray) mutations are distinguished from adenoma-specific and carcinoma-specific mutations (green and red, respectively). (**B**) For each lesion (gray and black for adenomas and carcinomas, respectively), the ratio of lesion-specific and lesion-common mutations are shown (log-scaled; *y*-axis). Dominance of lesion-specific mutations over lesion-common mutations is shown as positive log-ratios. Asterisks indicate the three hypermutated cases. (**C**) cancer cell fractions (CCFs) are shown as mutation allele frequencies adjusted for estimated tumor purity and ploidy levels. Ad-Co and Ca-Co mutations are lesion-common mutations of adenomas and carcinomas, respectively. Ad-Sp and Ca-Sp mutations are for those of adenoma- and carcinoma-specific mutations.

Somatic mutations were identified by comparing the tumor-derived whole-exome sequencing data (adenoma or carcinoma) with those of matched normal data to subtract germline variants. We first investigated the abundance of somatic mutations (i.e., the number of exonic single nucleotide variants—SNVs—and insertions/deletions—indels) (Figure 1).

Somatic mutations identified in these 21 cases are detailed in Table S2. In 11 colorectal cancers (C1–C11), 90–237 and 75–163 mutations were observed for adenomas and carcinomas, respectively, except for a hypermutated case with 2434 and 2495 mutations in the adenoma and carcinoma (C4; Figure 1A). In 10 gastric cancers (S1–S10), 53–204 and 85–160 mutations were observed for adenomas and carcinomas, respectively, while two hypermutated cases had 924/982 (adenoma/carcinoma; S4) and 1148/1221 mutations (S10). Both tumor types did not show statistically significant differences in the mutation abundance between matched adenomas and carcinomas (*p* = 0.336 and *p* = 0.684 for colorectal and gastric cancers, respectively; paired *t*-test).

We also examined the ratio of lesion-specific mutations (i.e., adenoma- or carcinoma-specific) to lesion-common mutations (i.e., those observed both in adenomas and carcinomas) (Figure 1B). This ratio was not significantly different between paired adenomas and carcinomas (adenoma vs. carcinoma; *p* = 0.180 and 0.09 for colorectal and gastric, respectively; paired *t*-test). However, we observed a significantly higher ratio in gastric cancers compared to colorectal cancers, representing a dominance of lesion-specific mutations over lesion-common mutations (colorectal vs. gastric, *p* = 0.015; Welch’s *t*-test). In addition, we observed that the majority of mutations in the three hypermutated cases are lesion-common mutations, as indicated by negative log ratios (C4, S4, and S10; asterisks in Figure 1B). This suggests that mutation bursts in these cases are likely to have occurred before the divergence of malignant cells from the benign lesions. Next, we examined the cancer cell fraction (CCF) of mutations as a measure of clonality [12] (Figure 1C). Lesion-common mutations show a higher CCF compared to lesion-specific mutations, indicative of their enrichment for clonal mutations over subclonal mutations (*p* < 2.2 × 10^−16^ both for colorectal and gastric cancers; *t*-test). We also note that the CCF of adenoma-specific mutations is significantly lower than that of carcinoma-specific mutations (*p* = 0.044 and *p* = 0.016 in colorectal and gastric cancers, respectively). The higher CCF of carcinomas may be a consequence of clonal selection, e.g., selective sweeps or other evolutionary forces that are operative during malignant transformation. In addition, we examined the proportion of somatic mutations leading to amino acid changes, as well as the six mutation spectra (Figure S2). The proportions of different mutation categories in lesion-common mutations were compared to those of adenoma- and carcinoma-specific mutations, but no statistically significant difference was observed. This suggests that the mutation forces associated with an impact on encoded proteins and sequence contexts are largely similar throughout the adenoma–carcinoma transition in colorectal and gastric cancers.

### 2.2. Genes Enriched to Regional Mutation Classes

**Figure 2:**
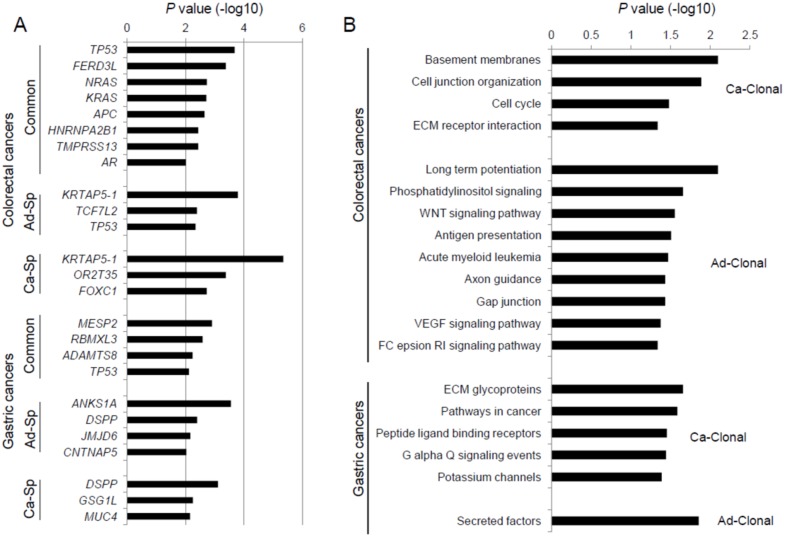
Recurrent mutations in region-specific classes. (**A**) Genes substantially enriched with three regional mutation categories (*p* < 0.01; MutSigCV) are shown for colorectal and gastric cancers. Common (lesion-common) mutations are shown with Ad-Sp and Ca-Sp (adenoma- and carcinoma-specific, respectively). (**B**) Gene sets are shown for those enriched with Ca-Clonal and Ad-Clonal mutations (those CCF_carcinoma_ > CCF_adenoma_ and CCF_carcinoma_ < CCF_adenoma_, respectively).

We identified recurrent mutations using MutSigCV [13] with respect to the regional mutation classes (i.e., common and adenoma-/carcinoma-specific mutations). Genes substantially enriched for mutations in three mutation categories are shown (MutSigCV *p* < 0.01; Figure 2A). In colorectal cancers, eight genes enriched with lesion-common mutations include known cancer-related genes involved in the colorectal adenoma–carcinoma sequence, such as *APC*, *KRAS*, and *TP53*. For example, *APC* mutations occur early in the dysplastic epithelium and are followed by mutations in *KRAS* and *TP53* during the dysplasia–adenoma and adenoma–carcinoma sequences, respectively [5,6]. We note that *TP53* mutations were also observed as adenoma-specific mutations (six common *TP53* mutations along with two adenoma-specific *TP53* mutations). Two adenoma-specific *TP53* mutations (one frameshifting indel/p.Pro169fs mutation and one missense/p.Leu125Pro mutation in C2 and C4, respectively) occurred in the adenomatous lesions after the emergence of malignant clones. This observation is consistent with the previously assumed temporal sequence of mutations, in which *TP53* mutations arise between the late adenoma and carcinoma stages [5,6]. However, it also implies that these *TP53* mutations may not necessarily be associated with the emergence of malignant clones.

Recent investigation on colon adenoma–carcinoma samples has shown that *APC, TTN, TP53, KRAS*, *OBSCN*, *SOX9*, *PCDH17*, *SIGLEC10*, *MYH6*, and *BRD9* may represent early drivers in colorectal carcinogenesis. Furthermore, *AMER1* and *PRICKLE2* mutations were classified as early driver genes along with *APC* and *SOX9* associated with dysregulation of Wnt signaling [14]. Compared with lesion-common mutations in this study, however, no overlaps of mutations were observed beyond the *APC*, *KRAS*, and *TP53* mutations.

Among the common mutations in colorectal cancers, *NRAS* mutations were frequent (p.Glu61Lys, p.Glu61Arg, and p.Glu61Leu missense/common mutations in C1, C3, and C7, respectively), although this event has been previously reported to be relatively rare (<5%) in colorectal cancers [15]. This suggests that *NRAS* mutations may represent early genomic events similar to *KRAS* mutations, but further investigation is required to determine whether *NRAS* mutations are more frequent in a wider context or just limited to the unique settings of our study (carcinomas arising from colorectal adenomas). Mutations in *TMPRSS13* have been previously identified in colorectal adenomas [16], and their regional presentation in our study (lesion-common mutations) indicates that *TMPRSS13* mutations in colorectal cancers can be early events like *APC* or *KRAS* mutations. *TCF7L2* mutations have been frequently observed in colorectal cancers as various types of genomic aberrations including mutations, gene fusions, and chromosomal deletions [3]. We observed that *TCF7L2* mutations (one splicing and one frameshift indel) were adenoma-specific, so the oncogenic roles of *TCF7L2* mutations may not be related to malignant transformation. Some recurrent, lesion-specific mutations were in-frame indels observed in *KRTAP5-1* and *FOXC1*. Although the functional relevance of in-frame indels is elusive, it has been previously proposed that the upregulation of *FOXC1*-encoded transcription factor is known to induce epithelial to mesenchymal transition [17] as a potential marker for malignant progression and metastasis [18]. Interestingly, Lin et al. revealed that a series of genes including *APC*, *TMPRSS13*, *CTNNB1*, *TCF7L2*, and *TP53* were frequently mutated in conventional colorectal adenomas, which is consistent with our study findings [16].

In gastric cancers, we observed *TP53* mutations as lesion-common-enriched mutations. Previous study with gastric adenoma–carcinoma pairs identified recurrent mutations of *TP53*, *APC*, *RNF43*, and *RPL22* in synchronous adenomas–carcinomas as lesion-common mutations [19], and the difference of mutation profiles may be partly because of the histologic difference between studies (e.g., synchronous or co-existence of benign and malignant lesions). Lesion-specific mutations in *JMJD6*, *CNTNAP5* (adenoma-enriched), and *GSG1L* (carcinoma-enriched) were also identified.

We performed CCF or clonality-based gene set enrichment analysis for lesion-common mutations. CCF is a measure of the fraction of cells harboring a mutation and has been used to classify mutations as clonal or subclonal [12]. The changes in CCF between adenomas and carcinomas may indicate the potential functionality of the mutations. For example, the mutations that can drive malignant transformation arising in the adenoma may be present as subclonal and clonal mutations in paired adenomas and carcinomas, respectively. Thus, we calculated the difference in the CCF between paired lesions (CCF_carcinoma_ − CCF_adenoma_) for common mutations and performed a preranked version of GSEA (Figure 2B). As a result, we observed that mutations in genes involved in the extracellular matrix (ECM) had an elevated CCF in carcinomas compared to matched adenomas (Ca-Clonal; CCF_carcinoma_ > CCF_adenoma_). Of note, ECM-related functions were also observed in Ca-Clonal mutations in gastric cancers. Ad-Clonal genes (CCF_carcinoma_ < CCF_adenoma_) in colorectal cancers largely represented signaling pathways, including the phosphatidylinositol, Wnt, and VEGF pathways. This clonal change of mutations may indicate that the malignant transformation represents a transit from aberrant signaling pathway-driven cellular proliferation to elevated invasive potential with aberrant ECM molecules. Clonality-based GSEA results are listed in the Table S3. We further performed GSEA of two cancer types combined with three hypermutated cases ignored (C4, S4, and S10), the most enriched functional category in clonal mutations was ”focal adhesion”, which is consistent with ECM-related functions (Table S3).

**Figure 3:**
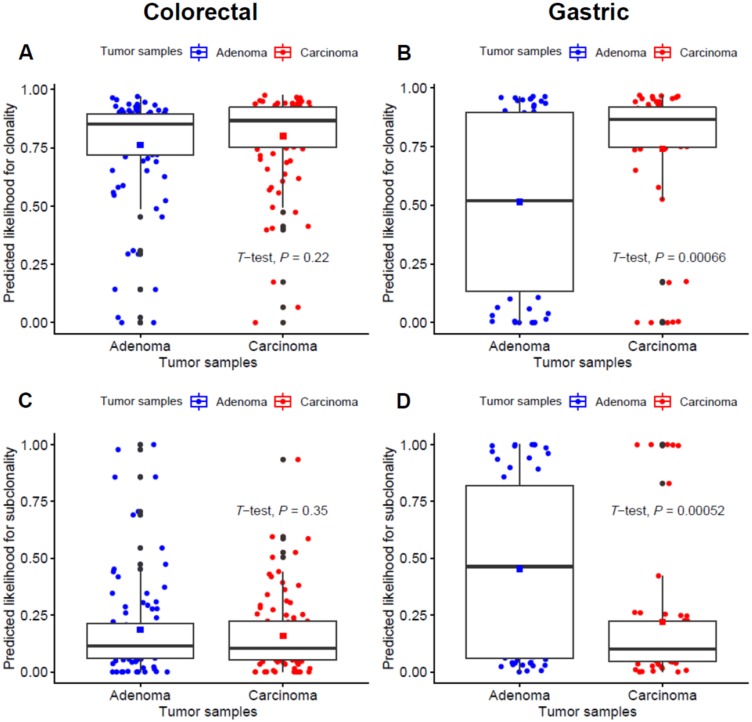
Comparison of clonality for tier 1 and 2 driver gene mutations between adenoma and carcinoma pairs. (**A**,**B**) the predicted likelihood for clonality in colorectal and gastric samples, respectively. (**C**,**D**) the predicted likelihood for subclonality in colorectal and gastric samples, respectively.

In addition, we conducted clonality analysis focused on driver mutations listed in the IntOGen database between adenoma–carcinoma samples (Figure 3). For tier 1 and 2 mutations representing functional cancer drivers, the predicted likelihood for clonality was significantly higher in gastric carcinoma samples (*p* < 0.001; Figure 3B), while the estimated subclonality was significantly higher in gastric adenoma samples (*p* < 0.001; Figure 3D). In line with this, there also seems to be a trend for higher clonality and subclonality in colorectal adenoma and carcinoma samples, respectively, although it did not reach statistical significance (*p* = 0.22 and 0.35 for the prediction for clonality and subclonality, respectively; Figure 3A,C).

For gastric samples, we found that the mutations in cancer driver genes are likely to be subclonal in adenomas but are clonal in carcinomas, suggestive of the functional roles of cancer driver mutations in gastric carcinogenesis. Meanwhile, the probability of mutations being clonal was not significantly different for colorectal adenoma–carcinoma pairs. The relative homogeneity of clonality across driver mutations in colorectal cancers suggests that driver mutations acquire clonality early, before the divergence of adenoma and carcinomas for these types of cancers. 

### 2.3. Mutation Clusters between Adenoma–Carcinoma Pairs

**Figure 4:**
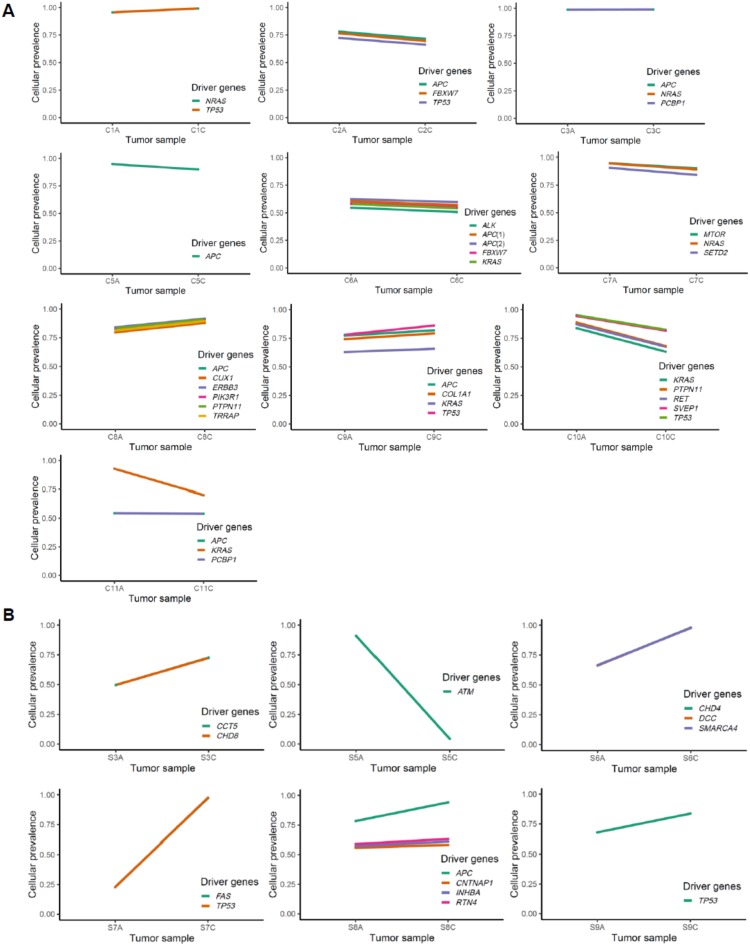
Cellular prevalence of mutation clusters using PyClone between adenoma and carcinoma pairs. (**A**) and (**B**) show the inferred cellular frequency of mutation clusters containing driver mutations in colorectal and gastric samples, respectively. The annotated genes (right) indicate the lesion-common mutated driver genes.

The change of clonality of driver genes during adenoma–carcinoma sequence was further investigated using PyClone. For the clusters containing lesion-common driver mutations obtained from PyClone analysis using the “two-sample” option, we compared the patterns of change in cellular prevalence, for example differential CCF levels between adenoma and matched carcinoma pairs (Figure 4A,B).

The value of CCF in ABSOLUTE and cellular prevalence in PyClone seems generally concordant because roughly PyClone can be considered to include an extra step of Bayesian non-parametric clustering to the ABSOLUTE-like procedure to reduce noises in predicting proportions of cells having the mutation.

In four colorectal cases (C1, C3, C8, and C9), we observed a minimal increase in the cellular prevalence of mutations during the adenoma–carcinoma sequence, and six colorectal cases (C2, C5, C6, C7, C10, and C11) showed a minimal reduction in the cellular prevalence of mutations during this same sequence, indicative of random fluctuations rather than changes having directions. On the other hand, for gastric cancers, all cases except one (S5) revealed clear increase in the cellular prevalence of mutations in carcinoma samples (*p* = 0.09). This finding is consistent with our results from clonality analysis, suggesting that not all driver mutations are associated with the emergence of malignant clones, especially in colorectal carcinogenesis.

In evolutionary biology, fitness landscapes (evolutionary landscapes)—a concept introduced in 1932—have been used to describe the relationship between genotypes and reproductive successes [20] where peaks represent adaptive genotypes and valleys for less fit genotypes.

Recently, Cross et al. revealed that colorectal adenomas may grow across an undulating evolutionary fitness landscape, whereas carcinomas show a sharp fitness peak that may reflect stabilizing selection [21]. In their study, the shape of the fitness peaks was assessed by SNV-based intra-tumoral heterogeneity quantification and phylogenetic analyses. Considerable variations in branch length suggest that mutations may be accumulated faster in some tumor regions, which can be incurred by selection on a new fitness peak, suggesting that adenomas are more heterogeneous than carcinomas, i.e., a broader fitness peak in colorectal adenomas.

**Figure 5:**
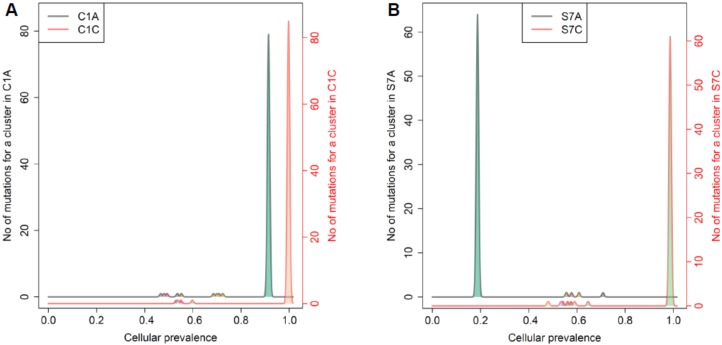
Inference of evolutionary fitness landscape using cellular prevalence of mutation clusters from PyClone analysis in representative colorectal and gastric tumors. The representative colorectal (C1) (**A**) and gastric (S7) (**B**) cases show higher peaks by numbers of mutations for a cluster (*y*-axis) and higher cellular prevalence with a smaller number of mutational clusters (*x*-axis) in carcinomas, suggesting stabilizing mutational selection during cancer evolution.

In this study, we have attempted to roughly outline the evolutionary fitness landscape of adenomas and carcinomas from Pyclone-based inference of clonal population structures. We compared each pair of adenomas–carcinomas in view of clonal clusters and cellular prevalence. The tumor with fewer clonal clusters and higher cellular prevalence compared to its pair can be considered as sharper fitness peaks, suggesting a tendency for evolutionary convergence. On the contrary, a rough fitness landscape consisting of a larger number of clonal clusters can result in multiple peaks, which tends to be evolutionary divergence. In this context, with clustering based on the estimated cellular prevalence and the number of mutations by PyClone analysis for each sample (i.e., “one-sample-only” option), we inferred the differences in the fitness landscapes within adenoma–carcinoma pairs (Figure 5A,B and Figure S3).

The analyses revealed that the majority of the cases (e.g., 7/11 colorectal cases; C1, C2, C3, C4, C7, C8, and C11 and 6/10 gastric cases; S3, S4, S6, S7, S8, and S9) showed sharper fitness peaks in carcinomas than in adenomas.

### 2.4. Copy Number Profiles

**Figure 6:**
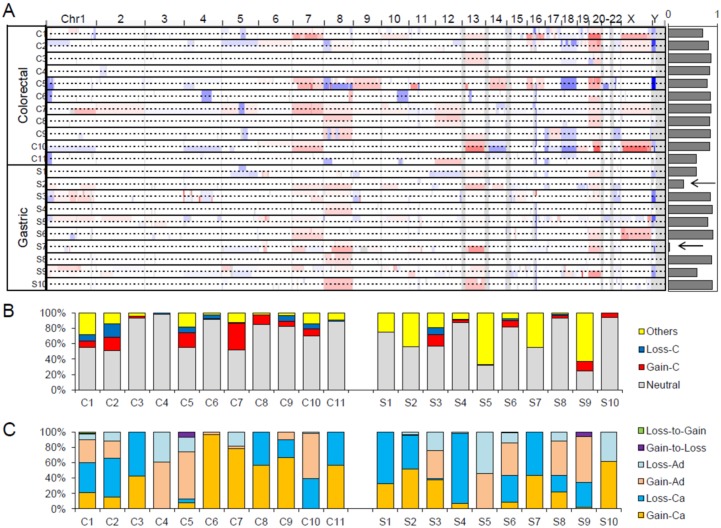
Somatic copy number alteration (SCNA) profiles of benign and malignant pairs. (**A**) Snapshot of Integrative Genomics Viewer (IGV) browser is shown as a genome-wide SCNA profile of 21 genomes examinations. The pairs of adenoma–carcinoma genomes are shown as pairs of individual lanes. The concordance level as the correlation between the segment level copy number ratios between the matched adenoma and carcinoma genomes are shown on the right. Arrows are for two cases with relatively discordant (correlation <0.5) cases. (**B**) The correlation (*x*-axis) and the case numbers (*y*-axis) are shown to demonstrate that these four cases are outliers from the majority of the cases. (**C**) Six classes of SCNA changes during the adenoma–carcinoma sequences.

The genome-wide somatic copy number alterations (SCNAs) profiles of 21 adenoma–carcinoma pairs are illustrated in Figure 6A. In the majority of cases, the SCNA profiles of adenomas and matched carcinoma genomes are largely concordant, as indicated by the high level of correlation between segment values of adenoma vs. carcinoma across the cases (Figure 6A; right). We noted two gastric cancers with relatively discordant SCNA profiles where the correlation of copy number segments between adenoma and carcinoma was less than 0.5 (arrows in Figure 6A). The discordance of these cases is largely due to the presence of carcinoma-specific SCNAs, and for these gastric cancers, a substantial number of SCNAs arise after the divergence of malignant tumor foci from the adenomatous lesion.

The observed copy numbers of primary tumor specimens are highly dependent on tumor purity and ploidy level, so the simple correlation of segment values is often misleading. To cope with this technical issue, we further identified absolute copy numbers (*n)* for individual copy number segments (i.e., copy number gains and losses for *n* > 2 and *n* < 2, respectively; *n* = 2 for neutral copy numbers). Genes were then intersected onto the copy number segments and further classified in the corresponding adenoma and carcinoma genomes. The relative proportion of genes in each case is shown in Figure 6B. The proportion of genes under segments with no copy number changes (Neutral; gray) as well as those in copy number gains and losses common in adenomas and carcinomas (Gain-C and Loss-C as clonal gains and losses; red and blue in Figure 6B, respectively) are shown with those under copy number changes between adenomas and carcinomas (Others). The relative proportions are variable across the cases, but we note that the proportion representing copy number changes between adenomas and carcinomas is higher in gastric cancers compared to colorectal cancers (*p* = 0.0275, *t*-test), suggesting that gastric cancer genomes acquire more SCNAs during the adenoma–carcinoma sequence. Figure 6C further demonstrates the six categories of copy number changes during the adenoma–carcinoma sequence, the extent of which is variable across cases.

### 2.5. Microsatellite Instability and Hypermutation

As a potential cause of hypermutated genomes, we examined microsatellite instability (MSI) using whole-exome sequencing data as previously described [22]. Two cases (C4 and S10) showed a substantial number of exonic MSI events, with significant (FDR < 0.05) DNA slippage events at exonic microsatellite repeats (26/75 and 423/506 exonic MSI events in adenoma/carcinoma, respectively). In contrast, the remaining cases showed fewer than five MSI events. These two cases were classified as MSI-H genomes. Deficiency in DNA mismatch repair leading to MSI-H explains two of the three hypermutated cases (C4, S4, and S10). Both genomes show characteristic length polymorphisms on frequent MSI targets such as *ACVR2A* (C4) and *TGFBR2* (S10) (Figure S4). The hypermutated case with no evidence of MSI-H (S4) may have other factors accounting for its elevated mutation burden. Since these genomes do not have mutations in genes known to cause hypermutation such as *POLD1* or *POLE*, further investigation is required to determine the potential cause of hypermutation.

## 3. Discussion

Serial biopsies of benign and malignant lesions from the same individuals were previously feasible only for rare clinical entities such as Barrett’s esophagus [23]. The current evolutionary studies therefore select a large number of adenomas and carcinomas from unrelated patients [16,21]. This cohort-level comparison may have limitations due to inter-patient heterogeneity.

Histological presentation of carcinomas arising from adenomas represents a unique clinical setting where benign and malignant lesions co-exist in the same individuals. Unlike synchronous presentation of multiple lesions of unclear origin, small malignant tumor foci are likely to have shared ancestry, having likely arisen in their embedded adenomatous lesions. Their apparent evolutionary relationship—ancestor adenomas and descendant carcinomas—provides an unprecedented opportunity for comparative analysis of benign and malignant genomes in the same individuals, enabling the following of adenoma–carcinoma sequences. Since DNA level alterations, including somatic mutations, may serve as evolutionary markers [24], we investigated the genetic concordance of adenoma–carcinoma pairs, as well as the mutation features and mutated genes associated with adenoma–carcinoma transition of colorectal and gastric cancers.

In this study, we observed a substantial level of mutation overlap between matched adenomas and carcinomas, i.e., 33.3–95.4%/46.6–93.0% of mutations in colorectal adenomas/carcinomas and 3.2–89.3%/3.4–83.9% of mutations in stomach adenomas/carcinomas were lesion-common mutations. This substantial overlap suggests that the examined adenomas and carcinomas share common evolutionary origins instead of representing synchronous lesions. However, it will require further investigation whether two lesions represent the results of stepwise evolution (i.e., the benign-to-malignant progression that does not accompany clonal sweep of adenomatous lesions) or parallel evolution where adenomatous lesions are heterogeneous in nature and only some benign clones possess the potential for malignant progression.

We also observed that the mutation abundance was not statistically different between matching benign and malignant lesions, leading to the conclusion that mutation abundance was largely comparable between the two lesions. Although the level of mutation abundance was relatively constant between adenoma–carcinoma pairs, analysis across the mutation categories with respect to mutation clonality measured by CCF revealed that the CCF was lower for adenoma-specific mutations compared to carcinoma-specific mutations. Given that the CCF measure reflects the level of heterogeneity in the subclonal mutation architecture, statistical differences in CCF values between adenoma–carcinoma pairs suggests that coexisting adenomas and carcinomas have been subject to different levels of evolutionary perturbation. There is evidence both against [25] and in favor of clonal sweep during cancer evolution [26]. Our results support the presence of clonal evolution during malignant transformation in the form of competition between major and minor subclones, instead of complete dominance by a single clone (selective sweep). In addition, clonality among tier 1 and 2 mutations was predicted to be higher in carcinomas than adenoma samples, suggesting that the known driver mutations have been subject to clonal selection, especially in gastric cancer evolution.

We observed a number of features indicating more accelerated evolutionary dynamics in gastric cancers than colorectal cancers. For example, the dominance of lesion-specific mutations over lesion-common mutations was more frequently observed in gastric cancers. It has been assumed previously that the ratio of relative abundance of lesion-specific and lesion-common mutations may reflect the extent of complexity in subclonal mutation architecture of cancer genomes with potential clinical implications [27]. The dominance of subclonal SCNA events was also noted to be frequent for gastric cancers, suggesting that gastric cancers may be more permissive to newly arising genomic aberrations, including somatic mutations and SCNAs, leading to the inter- and intra-tumoral heterogeneity of the disease [28].

Even for lesion-common mutations, the comparison of the CCF or clonality of mutations between adenoma and carcinoma may further identify mutations associated with malignant transformation. For example, mutations responsible for the malignant progression of benign adenomas would be expected to be present in a subclonal fraction of the precedent benign adenomas but become enriched in the progressed malignant carcinomas [11,26]. These subclonal-to-clonal mutations in adenoma–carcinoma pairs can be identified by comparison of their CCF values, and we identified a number of recurring molecular schemes (e.g., ECM) enriched with subclonal-to-clonal mutations across both tumor types. Along with the mutations enriched in regional mutation classes, these function-related mutations require further investigation for their roles in malignant transformation.

According to a study by Cross et al., the evolutionary trail across colorectal tumor cell populations appears to be a relatively flat fitness landscape for adenomas, in contrast to higher and sharper peaks for carcinomas [21]. In the present study, we have attempted to infer the evolutionary fitness landscape by comparing clusters and cellular prevalence between adenoma and matched carcinoma samples. We revealed that the majority of cases (seven colorectal and six gastric pairs) showed sharper peaks in carcinomas, representative of higher numbers of mutations per cluster and higher cellular prevalence with less mutational clusters. These findings are largely consistent with those of a previous study by Cross et al.

In the three hypermutated genomes identified in our study, the majority of mutations occurred as lesion-common mutations, suggesting the mutation burst leading to the mutator phenotype and their causal genomic events may occur early on in colorectal and gastric cancers. Two of the three hypermutated cases can be attributed to a deficiency in DNA mismatch repair, causing an elevated number of MSI events. Early studies have proposed that mutations of DNA mismatch repair genes may be early events, consistent with our observations [29,30]. One hypermutated case that could not be classed as a MSI-H genotype still showed an early mutation burst, but the cause of hypermutation was elusive. Given recent attention to hypermutation as a predictive marker for immune checkpoint blockade treatment [31], the potential cause and evolution of hypermutated genomes such as those described in our study requires further investigation.

## 4. Materials and Methods

### 4.1. Patient and Tumor Specimens

A total of 21 patients with early gastrointestinal cancer (11 colorectal and 10 gastric adenocarcinomas) and coexisting adenoma who had previously undergone endoscopic submucosal dissection in Seoul St. Mary’s hospital between 2013 and 2017 were enrolled. All cases were sporadic, without any familial history of colorectal or gastric cancer. Clinicopathological parameters, including age, sex, histologic type, tumor location, tumor size, and lymphovascular invasion status were reviewed retrospectively from the medical records and pathology reports. The patient information is summarized in Table S1. The study was approved by the Institutional Review Board of the Catholic University of Korea, College of Medicine (KC14TISI0436 and KC16SISI0960). All patients provided written informed consent before study enrollment. This study was conducted in accordance with the Declaration of Helsinki.

The formalin-fixed, paraffin-embedded tissues were cut and examined under a microscope by a pathologist specialized in gastroenterology. Figure S1 shows the representative hematoxylin–eosin (H&E) stained tissue sections from colorectal and gastric adenoma and carcinoma. Adenoma and carcinoma cells were selectively procured from H&E stained sections using a 30G1/2 hypodermic needle by microdissection. Purity of the tumor cells from the microdissection was approximately 70%. For control DNA, we used blood samples from each patient. For genomic DNA extraction, we used the DNeasy Blood and Tissue Kit (Qiagen, Hilden, Germany), following the manufacturer’s recommendation.

### 4.2. DNA Sequencing

Exonic DNA capture was done with the Agilent SureSelect Human 50 Mb kit (Agilent Technology), using genomic DNA from tumor pairs (adenoma–carcinoma) and matched controls (blood). Sequencing libraries were generated as per the manufacturer’s protocol, and sequencing was performed on an Illumina HiSeq2000 platform. For all libraries, 100 bp paired-end sequencing was performed, with an average target coverage of 99.07%. Table S4 summarizes the sequencing information.

### 4.3. Somatic Mutations

Sequencing reads were initially mapped on the reference sequence of UCSC hg19 human genomes using BWA (Burrows–Wheeler aligner) [32]. We used SAMtools [33] and Picard (http://broadinstitute.github.io/picard) to manage the sequencing data. The local realignment and score recalibration of sequencing reads was further performed using Genome Analysis ToolKit (GATK) [34]. Somatic single nucleotide substitutions (SNVs) and short insertions/deletions (indels) were identified from tumor and matched normal genome sequences using MuTect [35] and Indelocator [34], respectively. We also used ANNOVAR for functional annotation of somatic mutations on their encoded amino acids [36].

### 4.4. Copy Number Alterations

Somatic copy number alterations (SCNAs) were identified using VarScan2 [37]. Ratios of sequencing depth from the tumor and matched normal genomes were calculated for 1 kb bins, and guanine-cytosine (GC) -corrected ratios were segmented using the circular binary segmentation (CBS) algorithm [38]. Tumor purity and ploidy was estimated using the SCNA segment profiles and the allele frequencies of somatic mutations using the ABSOLUTE algorithm [39]. The cancer cell fraction (CCF) of somatic mutations as the proportion of cancer cells in the admixture cell population was also estimated using ABSOLUTE. CCF-based gene set enrichment analysis (GSEA) [40] was performed using c2cp (curated pathway) gene sets as available in MSigDB (http://software.broadinstitute.org/gsea/msigdb).

### 4.5. Microsatellite Instability

Microsatellite instability (MSI) was identified from the sequencing data as previously described [22]. A total of 146,447 exonic microsatellites were used as a reference. The length of sequencing reads from the tumor and matched normal genomes was identified for each exonic microsatellite. The distribution of repeat length between the tumor and matched normal genomes was compared per reference repeat, and the statistical significance was evaluated by Kolmogorov–Smirnov test.

### 4.6. Clustering of Tumor Cell Populations Using PyClone

To compare mutation-based subclonal structures between adenoma and carcinoma pairs, we used Bayesian clustering with PyClone (version 0.13.1) [41] to estimate per-cluster cellular prevalence based on somatic SNV and copy number using a Markov chain Monte Carlo-based (MCMC-based) framework with 10,000 iterations. Somatic SNVs and purity-adjusted allele-specific copy numbers were obtained from MuTect and ABSOLUTE, respectively, and used as inputs for PyClone. To identify the changes of the subclonal landscape between adenoma and carcinoma, we also ran PyClone using the “one-sample-only” option and assembled sample-wide results for individual patients. Serial analysis for shared mutations between adenoma and carcinoma was also performed using PyClone with the “two-sample” option to obtain changes of cellular prevalence of shared clusters.

### 4.7. Driver Mutation Identification

We employed the tier system of driver genes for colorectal and gastric cancers using the IntOGen database (catalog of driver mutations updated in May 2016, https://www.intogen.org/download). With tier 1 and 2 driver mutations from the IntOGen database, we performed PyClone clustering and CCF analysis between adenoma and carcinoma pairs [42,43].

Tier 1 driver mutations are those likely to be involved in oncogenic transformation with documented and reproducible activity relevant to cancer. Tier 2 mutations are composed of genes with strong indications of a role in cancer but considered to be of lower confidence as drivers. Tier 2 alterations are relatively more recent targets, where the body of evidence supporting their role is still being accumulated [44]. In this study, 74 and 58 genes (a list of genes is shown in Table S5) were obtained from the IntOGen database for the analysis of colorectal and gastric cases, respectively.

## 5. Conclusions

In summary, we report the genomic profiles of co-existing benign and malignant lesions that may provide the evolution-based categorization of genomic alterations and the inferred evolution of mutational landscape in colorectal and gastric cancers. In clinical practice, our results may aid in the discovery and development of the clinically important biomarkers in colorectal and gastric cancers.

## Supplementary Materials

The following are available online at https://www.mdpi.com/2072-6694/12/2/325/s1, Table S1, The clinicopathological information of 21 patients. Table S2, The list of somatic mutations identified in 21 cases. Case represent colorectal (C1–C11) and gastric (S1–S10) with “A” and “C” representing adenoma and carcinoma, respectively. Genomic coordinates of chromosome (Chr) and start/end points are based on hg19. Mutation allele frequencies are shown as TUMOR_ref and TUMOR_alt for read counts supporting the corresponding mutations and wildtype alleles. CCG represents cancer census genes. Table S3, The list of genes substantially enriched from CCF/clonality-based gene set enrichment analysis in colorectal and gastric tumors. Ca-clonal represents those enriched with CCF (carcinoma) > CCF (adenoma) and vice versa for Ad-Clonal. ES and NES represent enrichment score and normalized ES, respectively. “Common” tumor types represent the GSEA results of two tumor types combined excluded for three hypermutated cases. Table S4, The basic information of sequencing in 21 cases. Table S5, The list of tier 1 and 2 driver genes for analyzing colorectal and gastric cases. Figure S1, Representative histopathological micrograph of gastric and colorectal adenoma and carcinoma (H&E staining, magnification ×200). (A) colorectal adenoma, (B) colorectal carcinoma, (C) gastric adenoma and (D) gastric carcinoma. Figure S2, The proportion of somatic mutations leading to different amino acid changes as well as the six mutation spectra. The proportion of different mutation categories in lesion-common mutations were compared to those of adenoma- and carcinoma-specific mutations, but no statistically significant difference was shown. Colorectal (C1–C11) and stomach cases (S1–S10) are represented with C and S, respectively. Figure S3, Inference of evolutionary fitness landscape using cellular prevalence of mutation clusters from PyClone analysis per each tumor sample. (A) shows the inferred alteration of fitness landscape between colorectal adenoma–carcinoma pairs from C1 to C11, respectively. (B) reveals the inferred alteration of fitness landscape between gastric adenoma–carcinoma pairs from S2 to S10, respectively. The analyses revealed that the majority of the cases (e.g., 7/11 colorectal cases; C1, C2, C3, C4, C7, C8, and C11 and 6/10 gastric cases; S3, S4, S6, S7, S8, and S9) showed sharper fitness peaks in carcinomas. Figure S4, Examples of MSI events in two MSI-H cases. For 2 MSI-H cases (C4 and S10), their example MSI events in *AVCR2A* and *TGFBR2* loci are shown as the adenoma- or carcinoma-specific shortening of MS repeat length (*x*-axis) compared to their matched normals.
