# Supplementary material for: Mutation Analysis of Colorectal and Gastric Carcinomas Originating from Adenomas: Insights into Genomic Evolution Associated with Malignant Progression
Source: Cancers (Basel). 2020 Jan 31;12(2):325. doi: 10.3390/cancers12020325 (PMC7072232; doi:10.3390/cancers12020325)
Supplement: Supplementary file 1 [file cancers-12-00325-s001.zip › cancers-689749-supplement final/cancers-689749-supplementary material.pdf]

Supplementary Material

Mutation Analysis of Colorectal and Gastric Carcinomas Originating from Adenomas: Insights into Genomic Evolution Associated with Malignant Progression

Sung Hak Lee, Jinseon Yoo, Young Soo Song, Chul-Hyun Lim and Tae-Min Kim

Table S1. The clinicopathological information of 21 patients.

Tumor sample	Age	Sex	Histologic type/ Differentiation	Organ site	Tumor location	pT_stage	Cancer_size (cm)	SM_invasion depth (㎛)	Lymphovascular invasion
**S1**	44	M	tubular adenocarcinoma, well differentiated	Stomach	body & posterior wall	1a	1	NA	absent
**S2**	79	M	tubular adenocarcinoma, well differentiated	Stomach	antrum & lesser curvature, posterior wall	1a	2	NA	absent
**S3**	76	M	tubular adenocarcinoma, well differentiated	Stomach	antrum & posterior wall	1a	1.3	NA	absent
**S4**	81	M	tubular adenocarcinoma, well differentiated	Stomach	antrum & posterior wall	1a	3.4	NA	absent
**S5**	77	M	tubular adenocarcinoma, well differentiated	Stomach	body & greater curvature	1a	1.1	NA	absent
**S6**	54	M	tubular adenocarcinoma, well differentiated	Stomach	antrum & greater curvature	1a	1.7	NA	absent
**S7**	64	M	tubular adenocarcinoma, well differentiated	Stomach	antrum & lesser curvature	1a	1.3	NA	absent
**S8**	73	M	tubular adenocarcinoma, well	Stomach	antrum & greater	1a	1.2	NA	absent

			differentiated		curvature				
**S9**	61	M	tubular adenocarcinoma, well differentiated	Stomach	angle & lesser curvature	1a	2.9	NA	absent
**S10**	82	F	tubular adenocarcinoma, well differentiated	Stomach	angle & posterior wall	1a	2.1	NA	absent
**C1**	53	M	adenocarcinoma, moderately differentiated	Colorectum	transverse colon	1	1.5	600	absent
**C2**	54	M	adenocarcinoma, moderately differentiated	Colorectum	rectum	1	1.5	1000	absent
**C3**	65	M	adenocarcinoma, moderately differentiated	Colorectum	rectum	1	1	2000	absent
**C4**	64	M	mucinous adenocarcinoma	Colorectum	ascending colon	2	1	NA	absent
**C5**	68	M	adenocarcinoma, well differentiated	Colorectum	sigmoid colon	1	2.3	400	absent
**C6**	59	F	intramucosal adenocarcinoma, well differentiated	Colorectum	rectum	Tis	1.5	NA	absent
**C7**	71	F	adenocarcinoma, well differentiated	Colorectum	ascending colon	1	2.2	3500	absent
**C8**	80	M	intraepithelial adenocarcinoma	Colorectum	rectum	Tis	0.3	NA	absent
**C9**	72	F	intramucosal adenocarcinoma, well differentiated	Colorectum	cecum	Tis	3.7	NA	absent
**C10**	80	M	adenocarcinoma, moderately differentiated	Colorectum	rectum	1	1.3	1000	present
**C11**	66	M	intramucosal adenocarcinoma, well differentiated	Colorectum	rectum	Tis	4.3	NA	absent

Table S2–S5. Please view at the excel file.

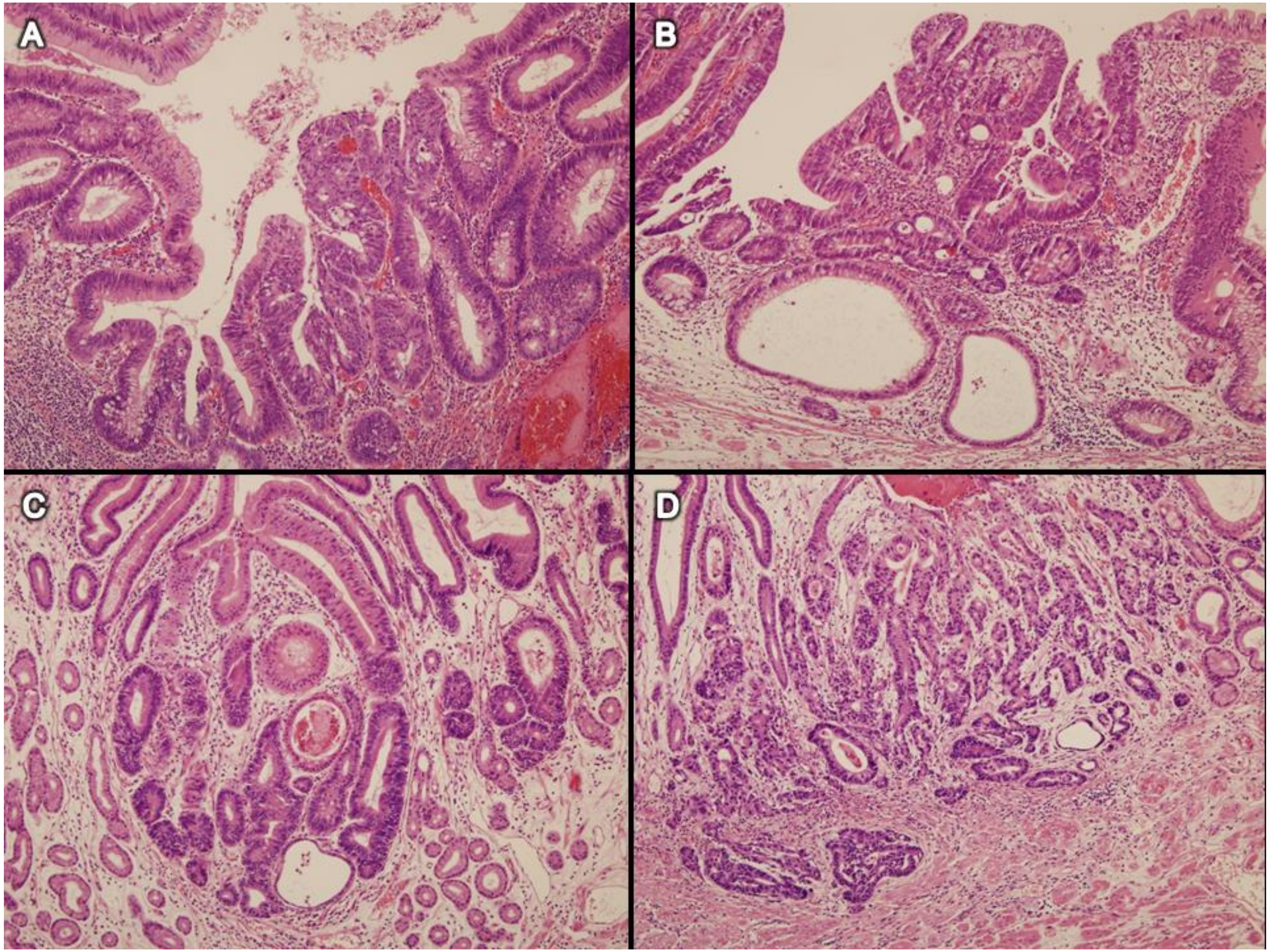

Figure S1. Representative histopathological micrograph of gastric and colorectal adenoma and carcinoma (H&E staining, magnification ×200). (A) colorectal adenoma, (B) colorectal carcinoma, (C) gastric adenoma and (D) gastric carcinoma.

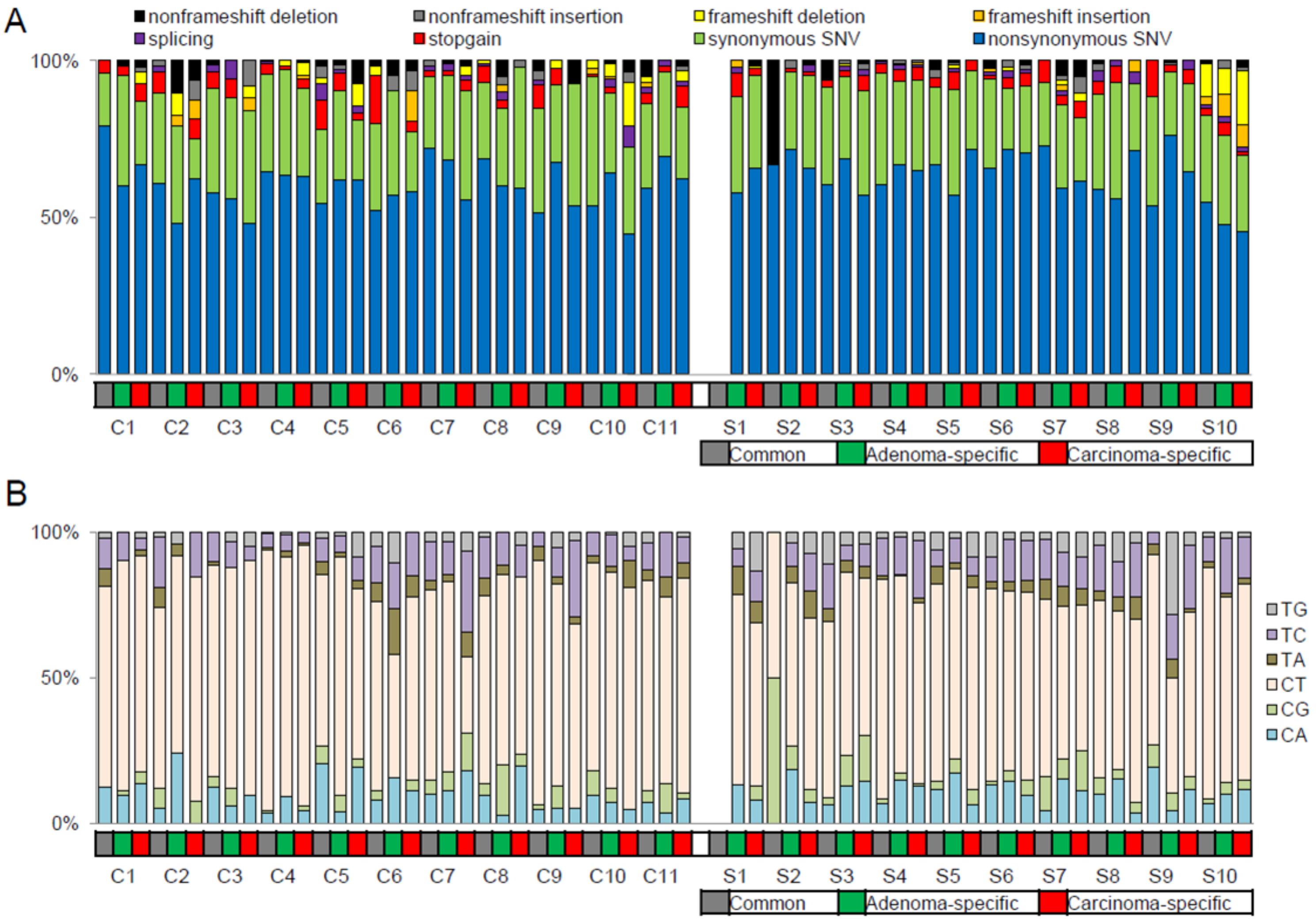

Figure S2. The proportion of somatic mutations leading to different amino acid changes as well as the six mutation spectra. The proportion of different mutation categories in lesion-common mutations were compared to those of adenoma- and carcinoma-specific mutations, but no statistically significant difference was shown. Colorectal (C1–C11) and stomach cases (S1–S10) are represented with C and S, respectively.

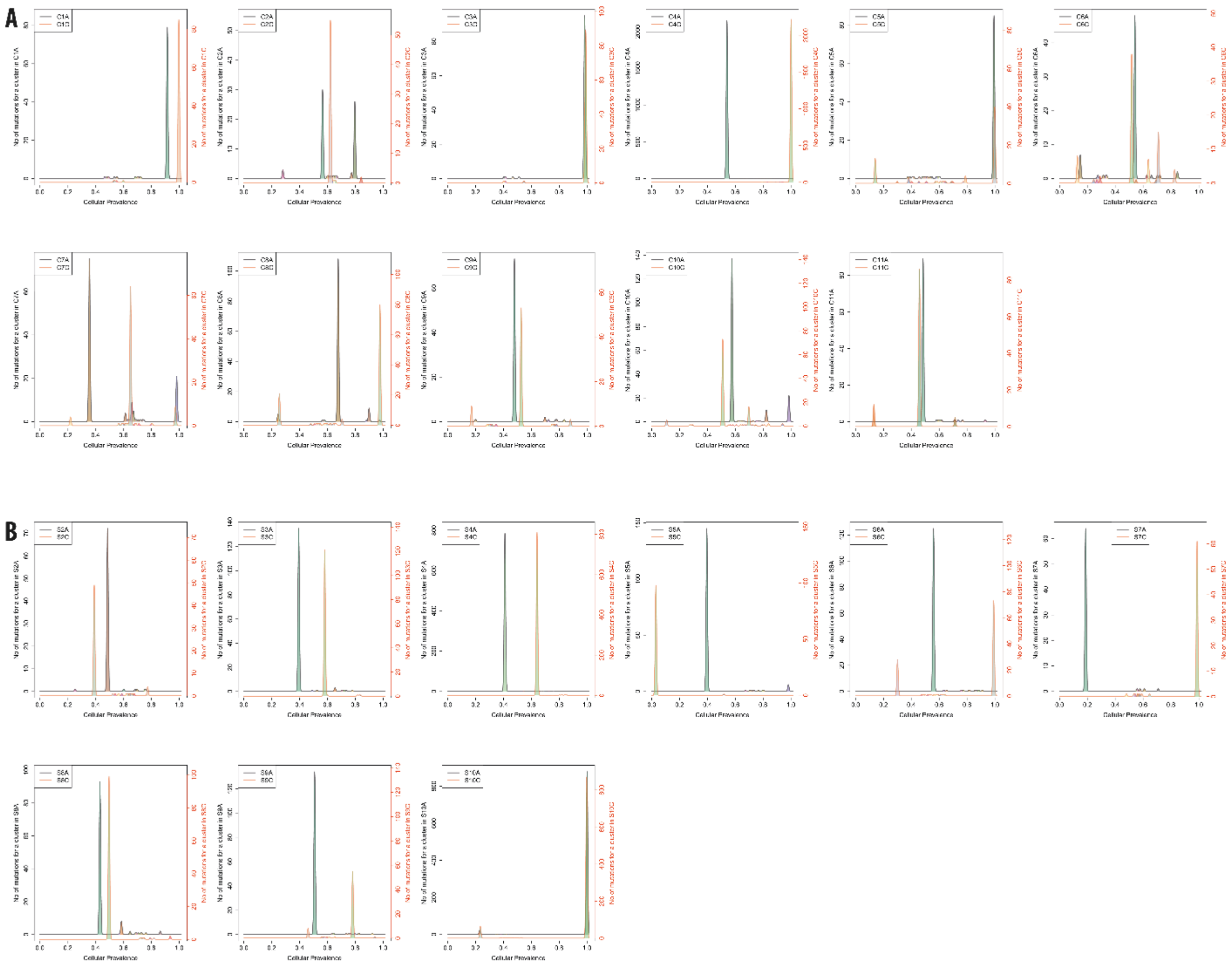

Figure S3. Inference of evolutionary fitness landscape using cellular prevalence of mutation clusters from PyClone analysis per each tumor sample. (A) shows the inferred alteration of fitness landscape between colorectal adenoma–carcinoma pairs from C1 to C11, respectively. (B) reveals the inferred alteration of fitness landscape between gastric adenoma–carcinoma pairs from S2 to S10, respectively. The analyses revealed that the majority of the cases (e.g., 7/11 colorectal cases; C1, C2, C3, C4, C7, C8, and C11 and 6/10 gastric cases; S3, S4, S6, S7, S8, and S9) showed sharper fitness peaks in carcinomas.

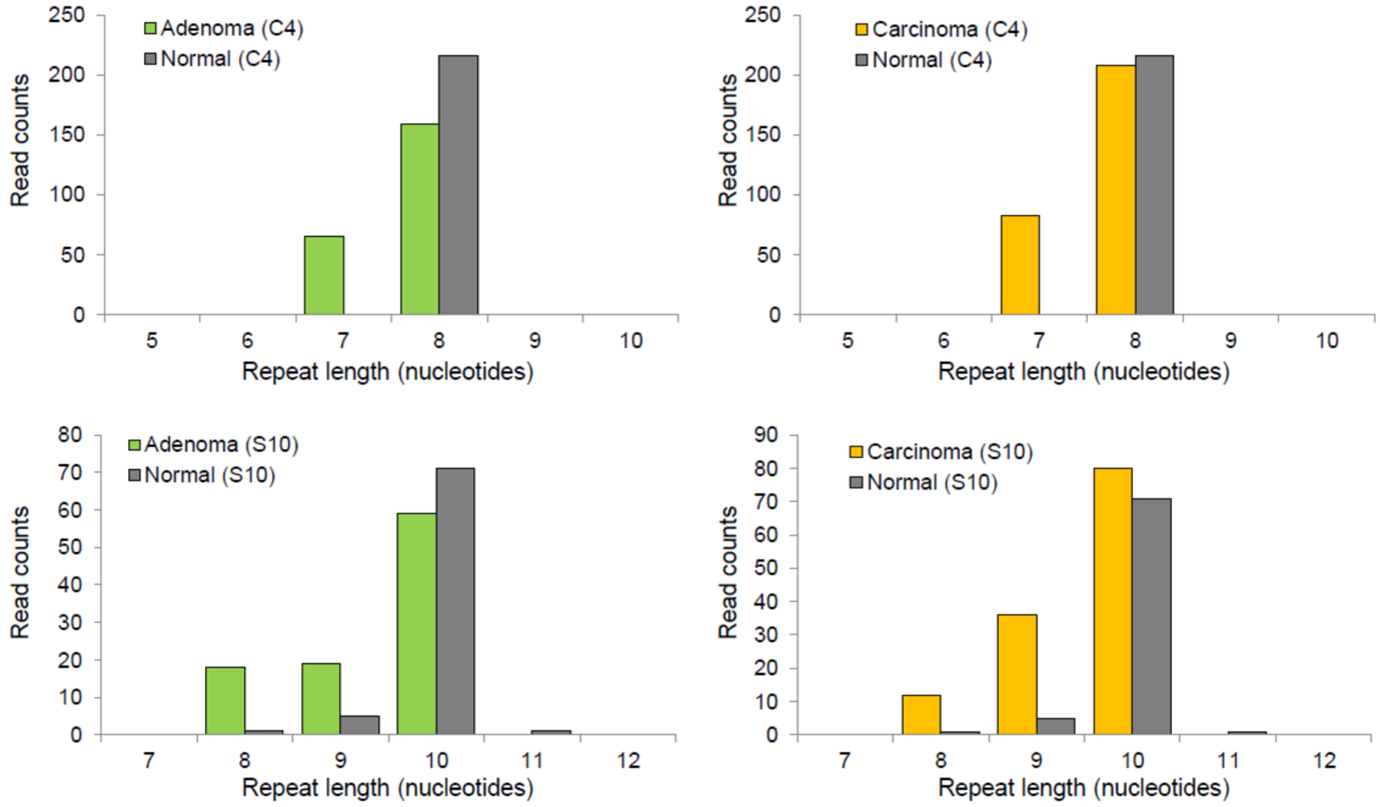

Figure S4. Examples of MSI events in two MSI-H cases. For 2 MSI-H cases (C4 and S10), their example MSI events in AVCR2A and TGFBR2 loci are shown as the adenoma- or carcinoma-specific shortening of MS repeat length (x-axis) compared to their matched normals.
